# MM-GradCAM: an improved multimodal GradCAM method with 1D and 2D ECG data for detection of cardiac arrhythmia

**DOI:** 10.1038/s41598-026-38654-w

**Published:** 2026-02-09

**Authors:** Fatma Murat Duranay, Ender Murat, Özal Yıldırım, Yakup Demir, Ru-San Tan, Niranjana Sampathila, U. Rajendra Acharya

**Affiliations:** 1 Department of Electrical and Electronics Engineering, Firat University, Elazığ, Turkey; 2 Department of Cardiology, Health Sciences University Gülhane Training and Research Hospital, Ankara, Turkey; 3 Department of Artificial Intelligence and Data Engineering, Faculty of Engineering, Fırat University, Elazığ, Turkey; 4 National Heart Centre, Singapore, Singapore; 5 Duke-NUS Medical School, Singapore, Singapore; 6 Manipal Institute of Technology, Manipal Academy of Higher Education, Manipal, 576104 India; 7 School of Mathematics, Physics and Computing, University of Southern Queensland, Springfield, Australia; 8 Centre for Health Research, University of Southern Queensland, Springfield, Australia

**Keywords:** ECG classification, GradCAM, Explainable AI (XAI), Cardiac disorders, Cardiology, Computational biology and bioinformatics, Diseases, Health care, Mathematics and computing, Medical research

## Abstract

As cardiac arrhythmia remains one of the leading causes of death worldwide, early and accurate diagnosis of cardiac arrhythmia is critical to improving patient outcomes. Electrocardiogram (ECG) analysis plays a critical role in the diagnosis of these diseases, and recent advances in deep learning have led to significant advances in automated ECG interpretation. However, the “black box” nature of these models limits clinical confidence and highlights the need for explainable artificial intelligence methods. This study presents an innovative MM-GradCAM method that combines two different data formats, providing explainability for both 1D ECG signal and 2D ECG image data. Using a dataset of more than 10,000 patients, a 17-layer CNN model capable of four-class arrhythmia detection was developed and separate explainability outputs were obtained for each data form. The resulting explainability maps were evaluated by a cardiologist and the interpretability and clinical significance of the model were verified. The signal form achieved 93.07% accuracy, while the image form achieved 97.44% accuracy. As a pioneering approach for explainability in medical diagnosis, MM-GradCAM has the potential to increase reliability and transparency in medical AI applications.

## Introduction

Arrhythmia is an important group of diseases in cardiovascular disorders and can occur on its own or with other cardiovascular diseases^1^. Arrhythmia can be represented by a slow, fast, or irregular heartbeat and can be grouped as life-threatening or non-life-threatening. In the diagnosis of arrhythmia, electrocardiography (ECG) is a commonly used method that records the electrical activity of the heart for a certain period of time^2^. Increased ease of use through digitization of ECG data and the development of algorithms to process the raw data could lead to significant improvements in automated ECG interpretation^3,4^.

The development of intelligent systems in the field of healthcare is very important, considering the processing of large amounts of raw data and the importance of the data they contain^5^. To compensate for visual errors and manual interpretation, researchers began to develop computer-aided design (CAD) systems to automatically diagnose ECGs^6^. In these systems, traditional methods design and select coded features, often by trial and error or by experience. Therefore, these systems tend to generate false positives that can lead to misdiagnosis and inappropriate treatment. Deep learning techniques have been developed to overcome these challenges faced by conventional systems and to provide better detection accuracy without the use of any hard-coded features^3,7,8^. In vital areas such as healthcare, the explainability of the outputs of deep learning models is critical to increase confidence in the model’s recommendations and to ensure acceptance among clinicians. There are many explainable artificial intelligence (XAI) approaches created for this purpose. One of the most popular of these approaches is Grad-CAM (Gradient-weighted Class Activation Mapping), which uses first-order gradients of the input signals^9^. This approach has been used to visualize the focal regions on ECG signals to provide clarity^10–15^. Thus, XAI approaches have increased the level of reliability and transparency as well as automation in medical diagnostics by making the decision processes of the models more understandable and interpretable.

The proposed method combines the explainability of two different data forms by adopting the basic principles of Grad-CAM approaches. Unlike the Grad-CAM methods used in other studies, the Multi-Modal GradCAM (MM-GradCAM) method we developed uses input data in both signal and image form. With this approach, signal-based explainability and pattern-based explainability obtained from time series are combined and a new perspective is brought to the literature. The contributions of our study to the literature are as follows:

- Using a large dataset of more than 10,000 patients’ ECG signals, a four-class arrhythmia detection was performed.
- ECG data were processed and recorded in both signal and image form, which were used together in the analyses.
- Convolutional neural network (CNN) models with 17 layers that can process both data forms were designed and the training of the models was carried out for 100 epochs.
- The trained models were evaluated on the same test patients and explainability outputs were obtained using the Grad-CAM method.
- The obtained explainability outputs were combined with the proposed new method and the results were interpreted by an expert cardiologist to assess the clinical validity of the study.

## Material and method

**Fig. 1:**
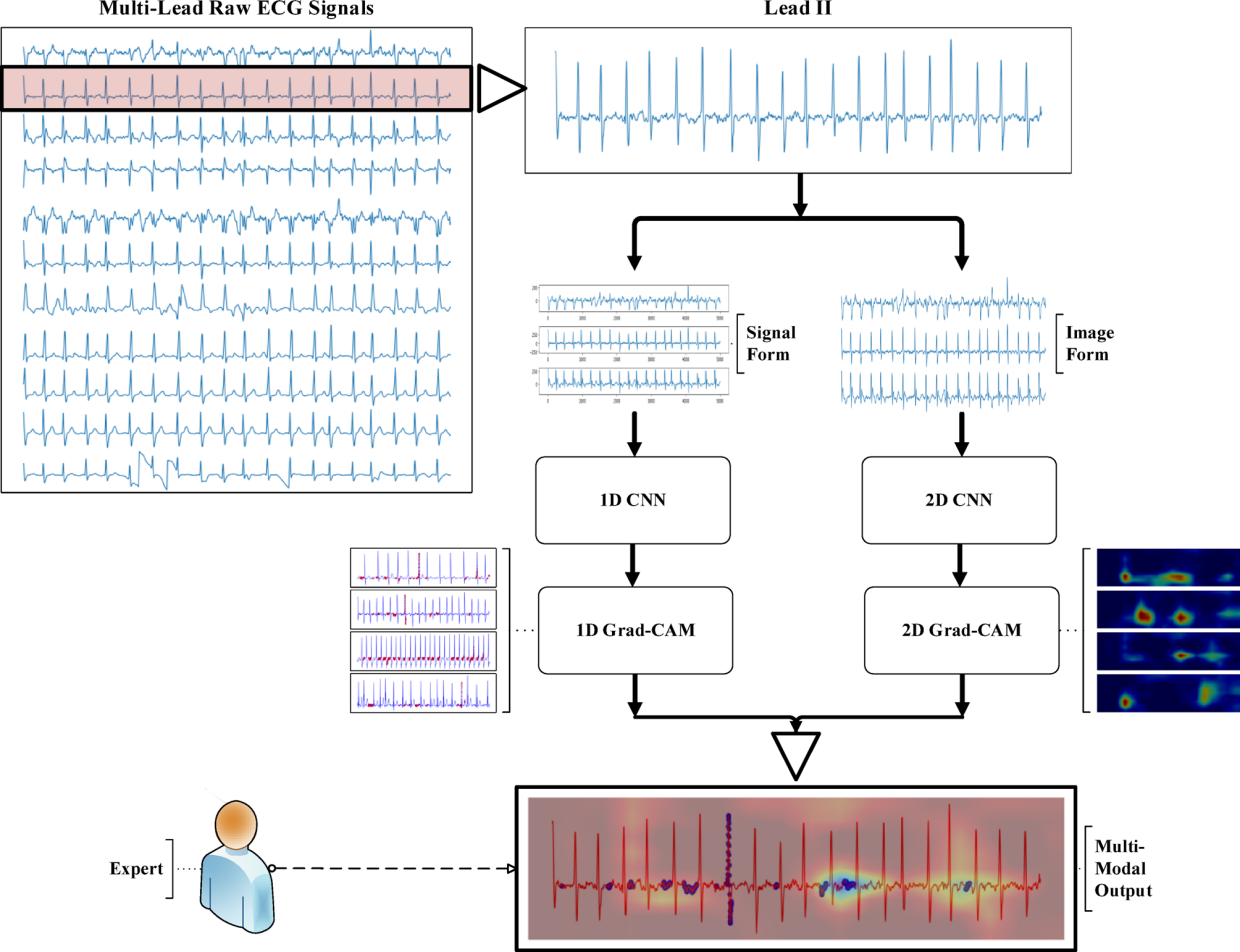
General flow representation of the study.

The general schematic representation of the method proposed in this study is presented in Fig. 1. In the first stage, only Lead II was selected from the multi-channel structure in the ECG data set because single-channel studies in the literature show that Lead II is widely used^16–20^. Following this selection, the single lead data were converted into two different forms and discrete operations were performed in parallel for each form. Customized models were designed and trained for each data form. Although the model architectures are kept similar in terms of a number of layers and ordering, the input dimensions of the 1D and 2D signals are different. Of note, the simple structure of the signal form and the relatively low data size enable fast deep model training processes. After the model training, output maps were created for each data form using the Grad-CAM method. Finally, these maps are combined to develop the proposed Multimodal-GradCAM (MM-GradCAM) method. The obtained explanations were interpreted in detail by an expert to evaluate the explainability of the method.

### Dataset and data processing

**Fig. 2:**
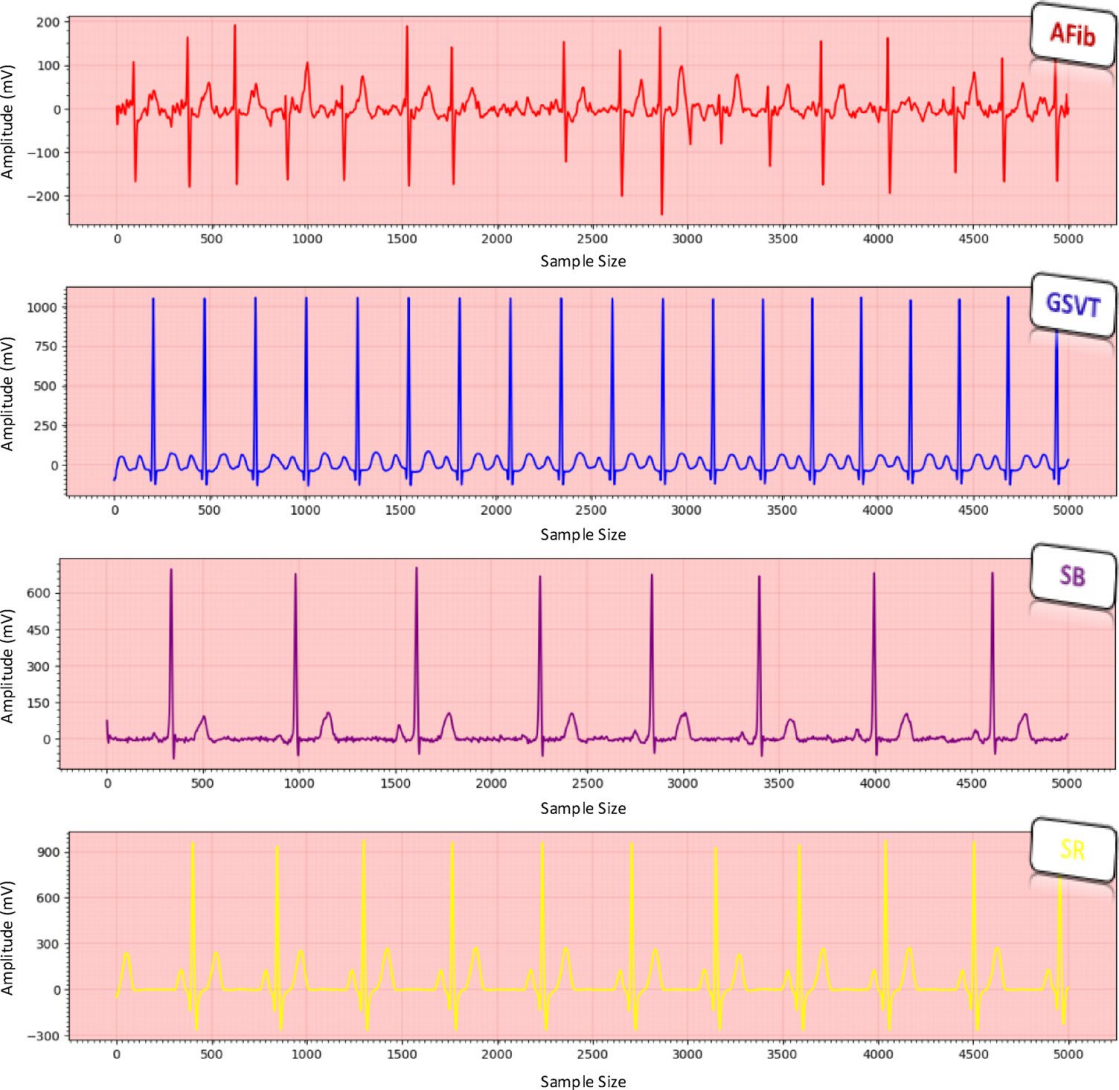
Examples of Lead II ECG signals from each arrhythmia class in the dataset.

The study dataset was derived from a large public database^21^, which comprises 10-s 12-lead ECG recordings of 10,646 subjects acquired at a sampling frequency of 500 Hz. The ECGs have been labelled by experts into 11 diagnostic classes, which can be merged into 4 classes: (1) “SB” includes sinus bradycardia; (2) “AFib”, atrial fibrillation and atrial flutter; (3) “GSVT”, supraventricular tachycardia, atrial tachycardia, atrioventricular node reentrant tachycardia, atrioventricular reentrant tachycardia and sinus atrium to atrial wandering rhythm; and (4) “SR”, sinus rhythm and sinus irregularity. The original dataset initially included data from 10,646 patients. However, after a thorough review, we excluded 58 patients due to incomplete or missing ECG signals, resulting in a final dataset of 10,588 patients for this study. For the current study, we used Lead II ECG signals—each with a 1D data dimension of 5000 (500 Hz × 10 s)—categorized into SB, AFib, GSVT and SR classes (Fig. 2). The authors did not define the four rhythm classes used in this study but directly inherited from the expert-labelled structure of the original Chapman dataset. Although the database contains 11 diagnostic labels, these labels are clinically consolidated into four major rhythm groups based on physiological similarity and real-world diagnostic practice. Therefore, the class selection follows the dataset’s standardized clinical mapping rather than an author-specific reduction. The novelty of the proposed method lies in the multimodal explainability framework, which is designed to be generalizable across different ECG rhythms, while the four-class structure provides a validated and balanced foundation to demonstrate its effectiveness.

#### Signal form

1D dimensional Lead II ECG signals from the multi-channel source data^21^ were input to the deep learning model without preprocessing. The integration of XAI requires customization of the signal form to facilitate annotability. For this purpose, the 1D Grad-CAM method was applied to input signal data of (10,588, 5000) dimensions using a 1D CNN model.

#### Image form

We used the Matplotlib library to convert Lead II ECG signals into 2D images. First, to enable XAI visualization, a plot of every signal data was created as a 300 × 600 figure. After plotting, graphic axes were turned off to reduce visual distractions, and marks on the x and y axes were removed to simplify the visuals, bringing the signal into focus. The results were saved as grey-scale 2D images to input into the model. Although incurring more processing power, storage and model training time costs, converting signals into image form will facilitate working with different methods, especially in the heat map generation phase.

### Classifier model

**Fig. 3:**
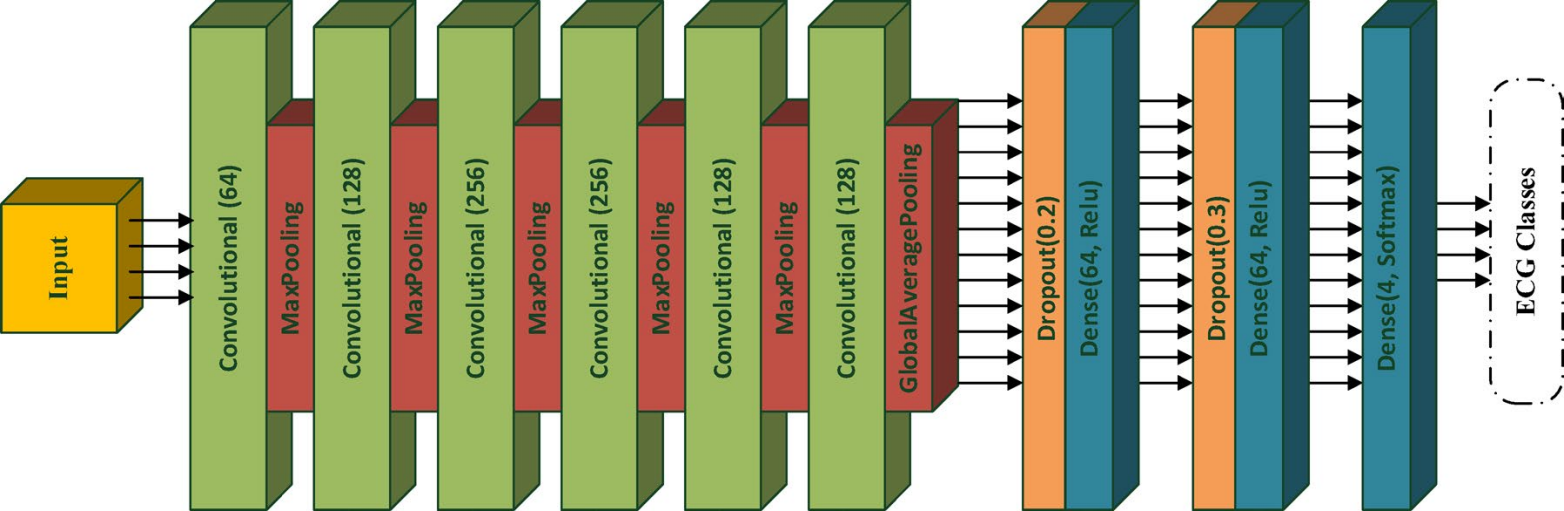
17-layer CNN-based model architecture.

We designed a 17-layer CNN model comprising six convolutional layers with 64, 128, 256, 256, 128, 128, 128- sized filters in sequential order. Max pooling layers are added after the first to fifth convolution layers and the global average pooling layer after the sixth. These are followed by a set of dropout and dense layers, repeated and connected to the final layer, a dense layer finished with a 4-class softmax classifier (Fig. 3).

This architecture was selected to balance representational capacity, computational efficiency, and generalization performance. The increasing number of filters in the early-to-mid layers enables the network to learn a hierarchical feature representation, starting with basic ECG waveform characteristics and progressing to more complex, rhythm-related patterns. The use of kernel sizes 3 and 5 in convolutional layers supports the extraction of short-range temporal dependencies, which are essential for capturing the morphology of P–QRS–T complexes. Max pooling operations with a pool size of 2 reduce temporal resolution, expand the receptive field, and prevent unnecessary parameter growth, while the GlobalAveragePooling layer significantly decreases the number of trainable parameters and provides structural regularization by summarizing each feature map into a single descriptor. The fully connected layers with 64 units, along with dropout rates of 0.2 and 0.3, were chosen to maintain a strong balance between learning capacity and robustness against overfitting. During optimization, the Adam optimizer with a learning rate of 0.0003 offered stable convergence and consistent validation performance. Overall, the architectural and training decisions were made based on empirical evaluations aimed at achieving optimal accuracy while maintaining a compact, generalizable, and computationally efficient model suitable for ECG-based arrhythmia detection.

**Fig. 4:**
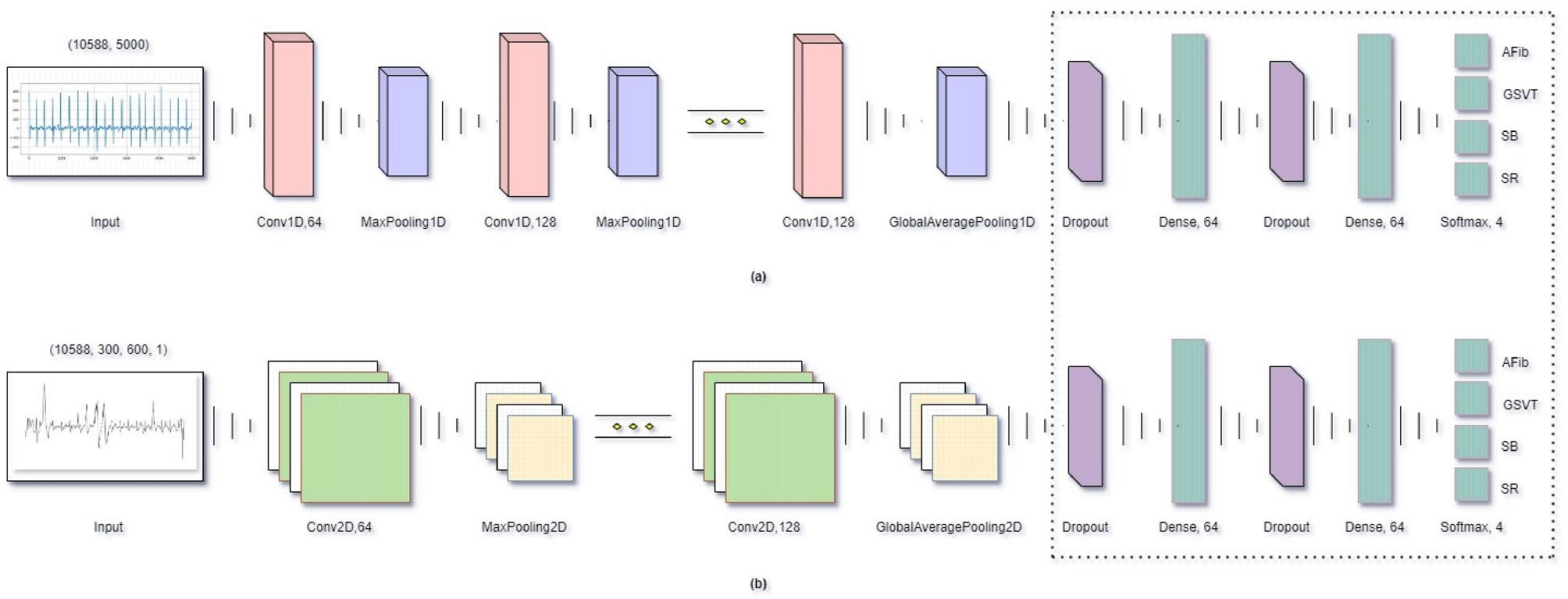
Model structure customized to 1D signal and 2D image inputs.

The CNN model was tweaked to accommodate the two different input data—(10,588, 5000) sized signal and (10,588, 300, 600, 1) sized image inputs—by adapting the convolution and pooling layers while keeping the base layer architecture and the number of filters in each layer constant, while the kernel shapes differ naturally between the 1D and 2D models. Conv1D layers and 1D pooling layers were used for 1D signal inputs; Conv2D layers and 2D pooling layers, for 2D image inputs. Dropout and dense layers were identical for both inputs (Fig. 4). To maintain comparable model capacity, the number of filters in each convolutional layer was kept the same across the 1D and 2D architectures. However, the kernel shapes naturally differ between 1D (k) and 2D (k × k) filters due to the dimensional characteristics of the input data.

### Explainability with GradCAM

GradCAM is a popular XAI technique that uses gradient information to calculate feature maps, facilitating visualization of model classification decisions. Applied to the final convolution layer of the target model, GradCAM generates a heat map showing the regions of concentration in the input, the densities of which allow experts to interpret the relative importance of information in the input data for the model. Many versions of GradCAM are available, but most have been applied to single data formats either 1D or 2D. We proposed MM-GradCAM, which is the first to combine the combined explainability of 1D signal and 2D image input formats.

#### Proposed MM-GradCAM explainability method

**Fig. 5:**
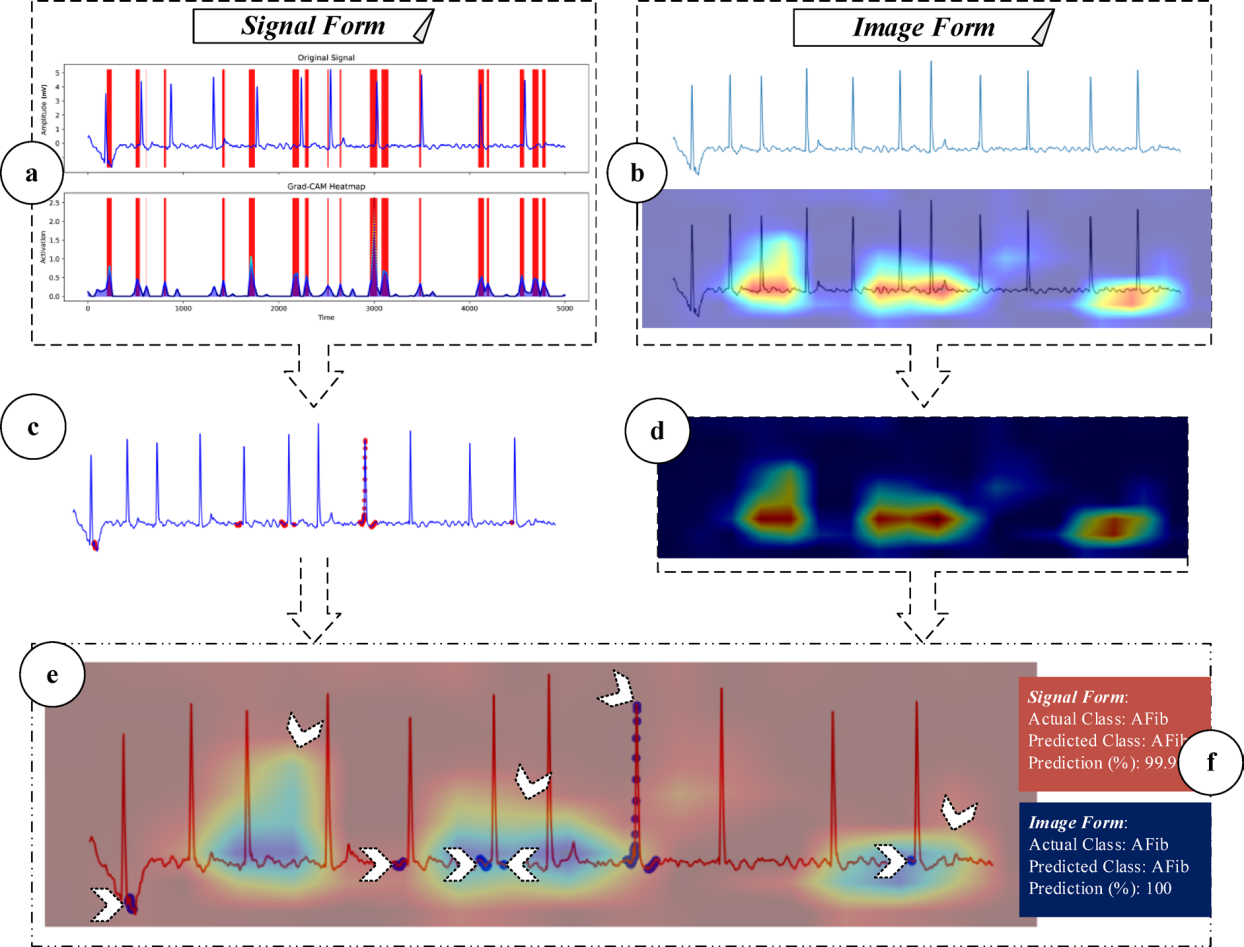
Flow diagram of the signal and image formats for the proposed MM-GradCAM method. In signal form, Grad-CAM applied to the last layer of the model generates output that highlights dense regions with red frames (**a**). These frames represent the dense regions taken into account by the final layer of the model and the densities of these regions are expressed with a certain value. According to a threshold value, the areas above this value are marked as red dots on the original signal (**c**). These heat maps are then separated from the original signal and presented in raw format (**d**). In image form, the Grad-CAM method applied to the images created from the raw signals generates heat maps of ECG images (**b**). By combining the outputs of both forms, both ECG time-series-based explainability and image-based explainability were provided on the same image (**e**). In addition, the softmax outputs of each sample are given together with the MM-GradCAM results and the classification success of the two forms are reported together (**f**).

MM-GradCAM was applied in parallel to the signal- and image-based CNN models. To integrate the 1D and 2D Grad-CAM outputs, a multi-stage fusion pipeline was implemented. First, the salient regions from the 1D signal-based Grad-CAM were extracted by threshold-based segmentation. In parallel, the 2D Grad-CAM heat maps were generated and normalized. Both outputs were then temporally aligned according to the original Lead II signal timestamps and spatially co-registered. Finally, the two modalities were merged into a unified MM-GradCAM map, allowing simultaneous visualization of time-series-based and pattern-based explainability. An example illustrating this integration is provided in Fig. 5. By combining the outputs of both forms, both ECG time-series-based explainability and image-based explainability, as well as their respective classification success, could be depicted on the same image.

## Training and experiments

### Environment

We created the CNN models in Python language using Keras and TensorFlow libraries and ran the experiments in a Google Colab environment on a personal computer equipped with AMD Ryzen 7 4800H, Radeon Graphics 2.90 GHz, and NVIDIA GeForce GTX 1660 Ti.

### Database

From our used 4-class study dataset of Lead II ECGs of 10,588 subjects, approximately 10% were randomly selected to be reserved for testing (Table 1).

**Table 1:** Patient data of the dataset.

Classes	Data split		
	Train data	Test data	Total data
AFib	1996	222	2218
GSVT	2038	222	2260
SB	3500	388	3888
SR	2000	222	2222
Total data	9534	1054	10,588

### Training arguments

Both models in signal and image formats were run with identical compilation settings. Adam optimization algorithm was used. The learning rate was set as 3 × 10^–4^, and the loss function, “categorical_crossentropy”. With accuracy as the performance metric, the models were trained for 100 epochs and the epoch in which maximum accuracy was reached in each training process was recorded.

### Testing of signal and image forms

**Fig. 6:**
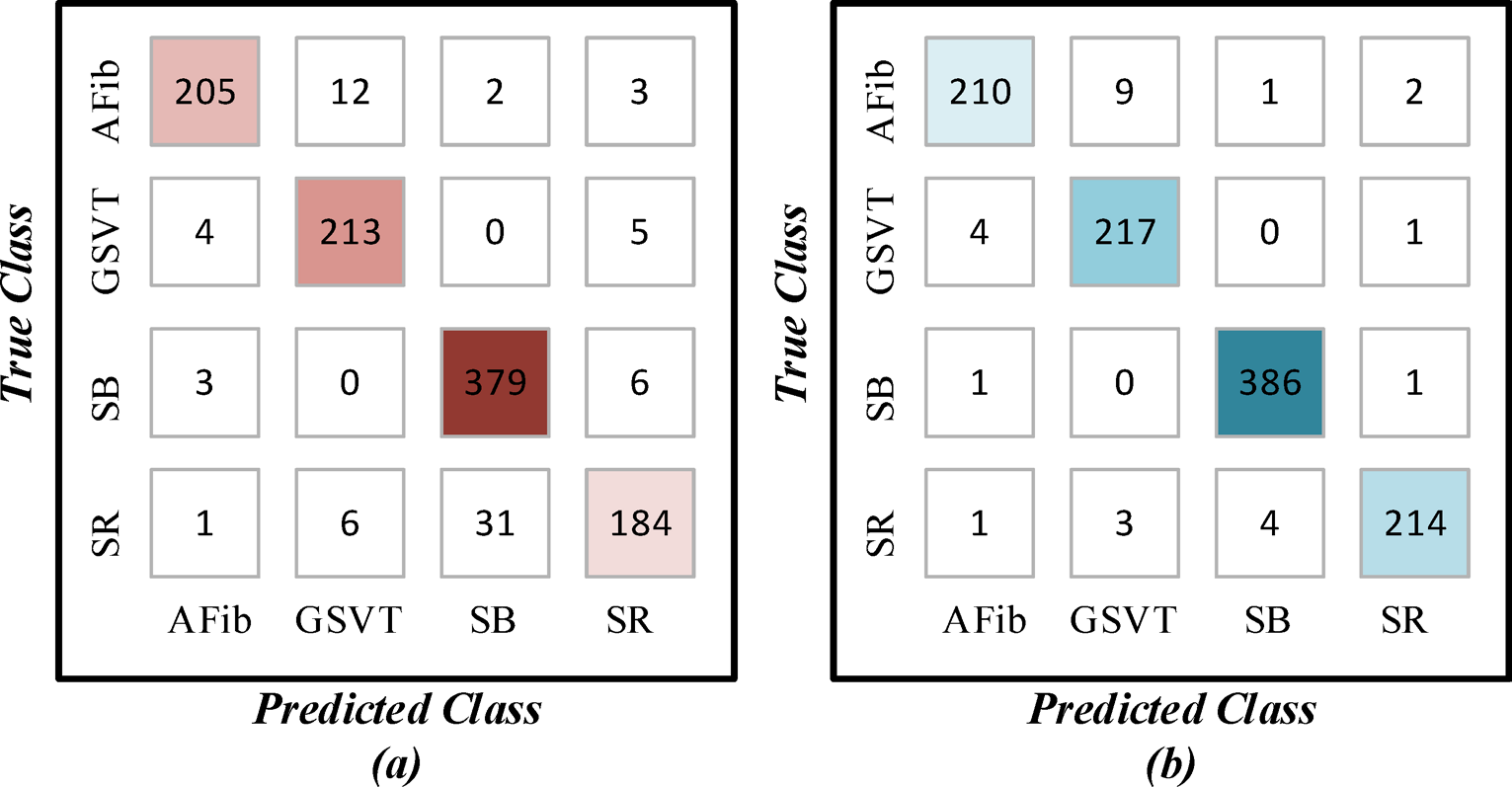
Confusion matrix for (**a**) signal and (**b**) image forms.

Trained models were applied in the testing phase to test the same unseen test dataset. While training the signal form during the testing phase took less time, the overall accuracy of the image form was superior compared with the signal form, 97.44% versus 93.07%. Table 2 summarizes the characteristics of the two forms and the detailed performance results. In the AFib class, both forms attained accuracy rates, albeit the image form performed better in terms of recall. In the GSVT class, the image form attained superior results across all evaluation metrics. In the SB class, the precision of the signal form was 0.92, 0.07 less than the image form. In the SR class, the signal form performed the worst across all classes, in contrast to the image form, which performed well. The confusion matrices for both forms are also given in Fig. 6.

**Table 2:**
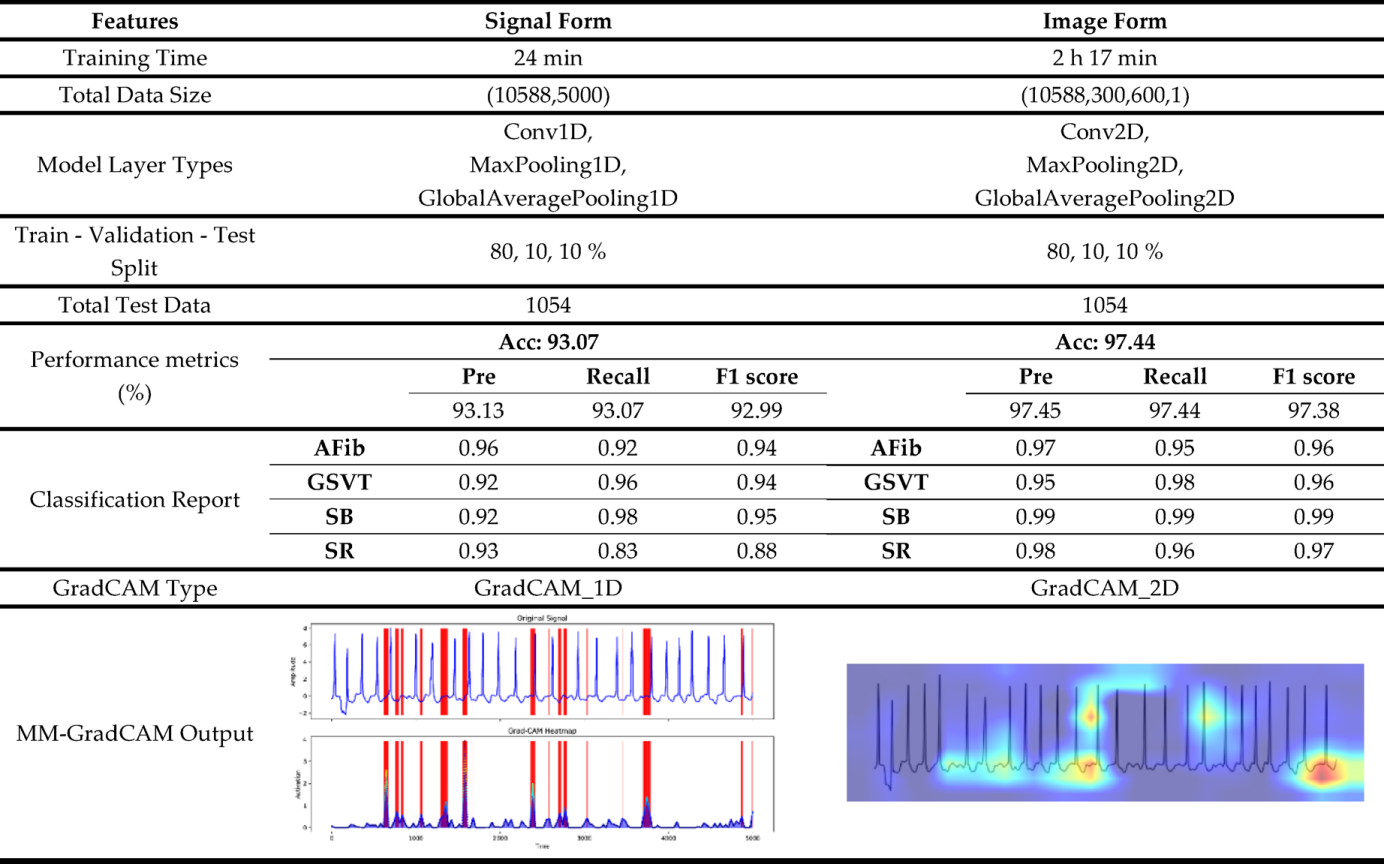
Comparison of the test phases of signal and image forms.

## Explainable results and expert assessment

**Fig. 7:**
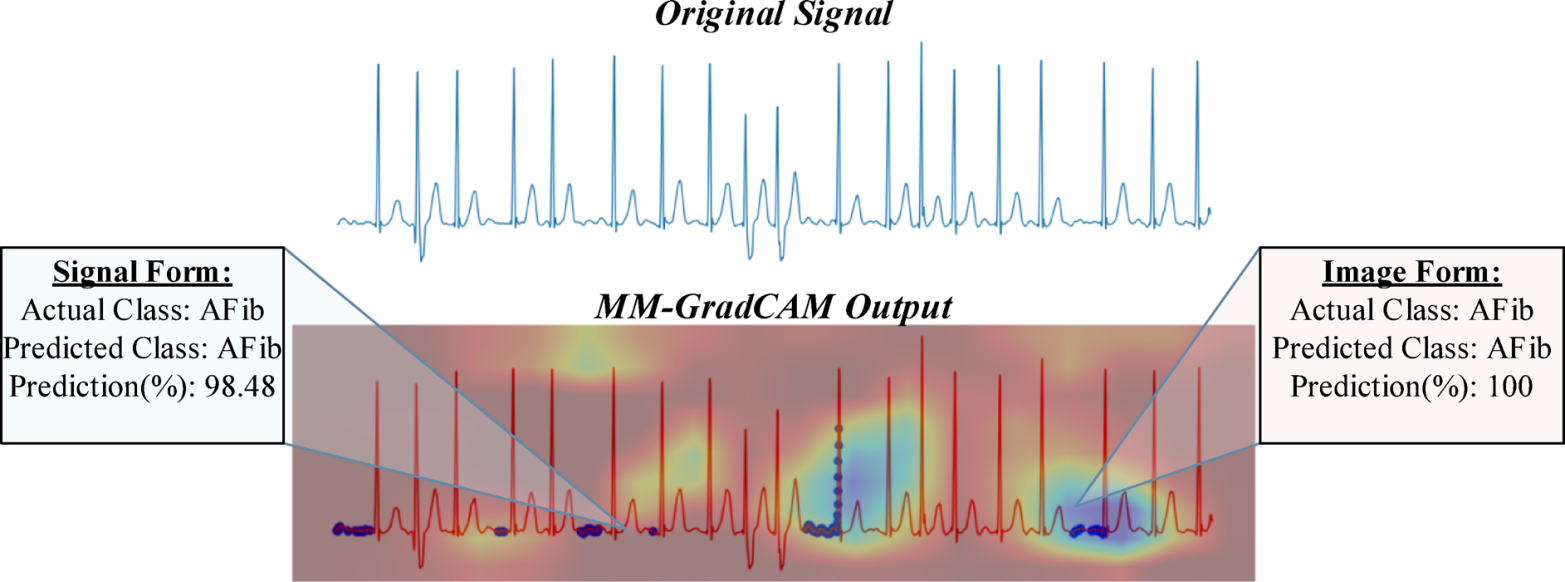
MM-GradCAM output of example Patient-1 belonging to AFib class. The regions where the signal form is focused largely correspond to the pre-systolic phases, where P waves are expected normally. The areas where the image form is concentrated are generally associated with increasing R-R intervals. At the intersection points of both forms, the absence of P waves and the relative increase in R-R intervals reinforce each other, thereby enhancing model explainability and robustness.

**Fig. 8:**
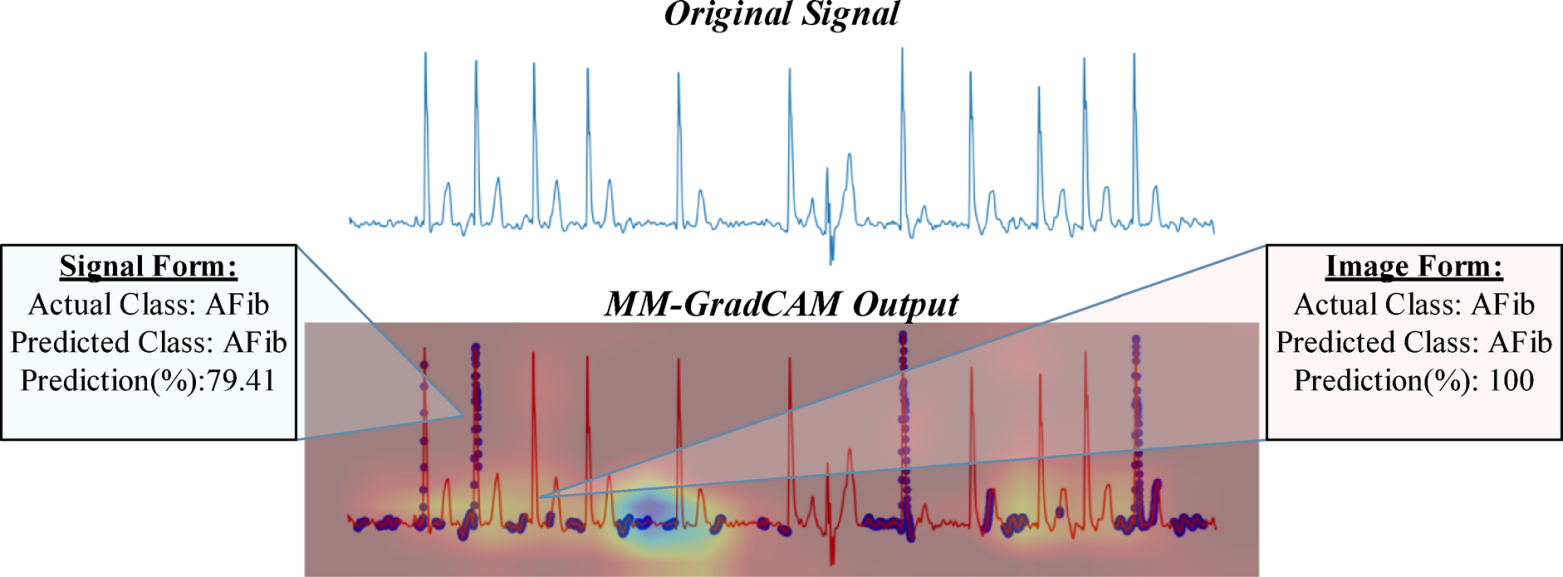
MM-GradCAM output of example Patient-2 belonging to AFib class. The signal form generally focuses on a few selected R waves with high voltages. This reduced sensitivity for R waves, in general, likely led to poor model performance for AFib detection. The image form focuses on the regions between the T wave and the QRS complex, where the absence of detected P waves may suggest AFib; this resulted in a higher accuracy for AFib detection.

**Fig. 9:**
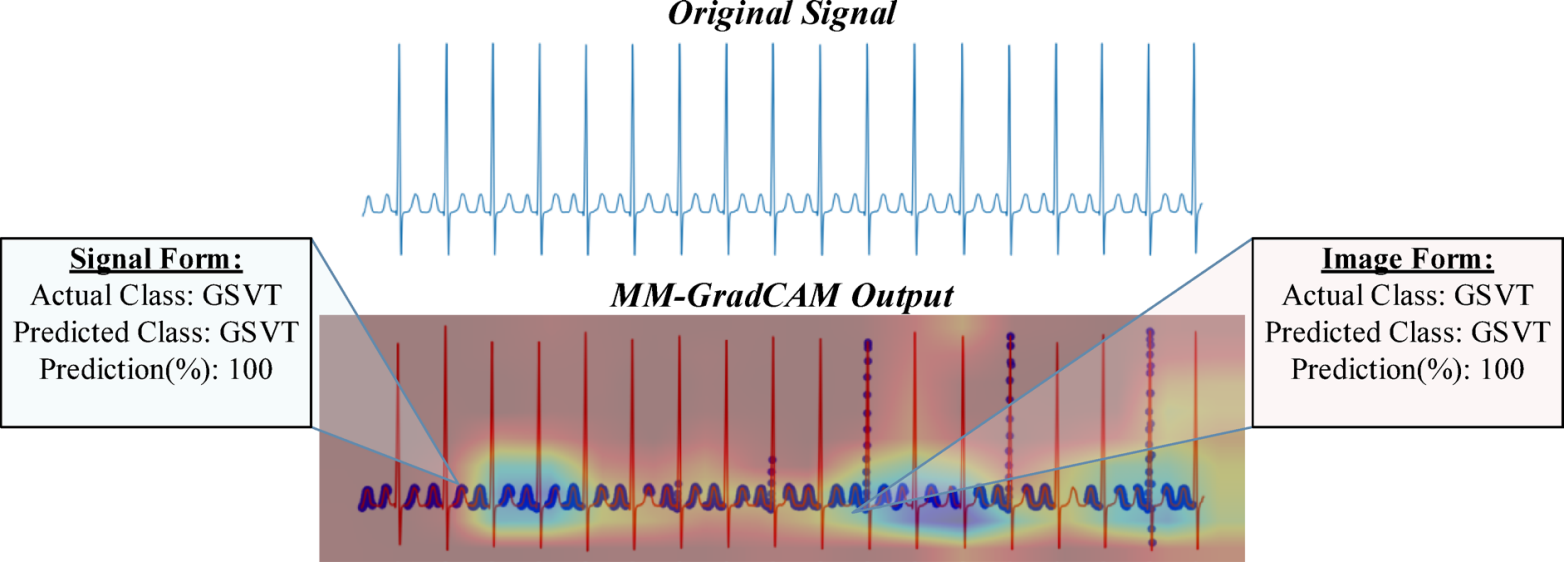
MM-GradCAM output of example Patient-3 belonging to GSVT class. In an example GSVT patient, the signal form is focused on almost all P and T waves, allowing the interpreter to conclude that the pulse is regular and rapid. While useful here, not all GSVT have prominent P waves. In contrast, the image form concentrates on separate discrete areas, running counter to the generalized nature of morphological ECG manifestations associated with GSVT. Here, the behavior and output of the signal form constitute a more logical conclusion and better explainability.

**Fig. 10:**
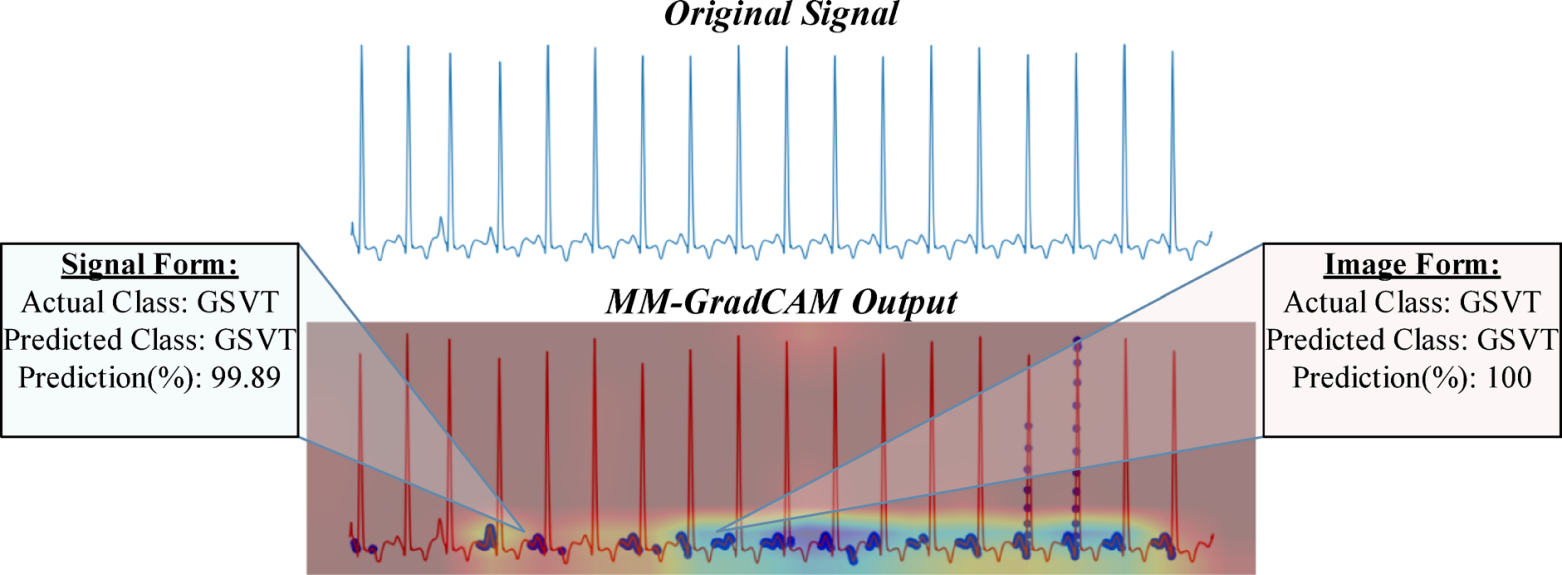
MM-GradCAM output of example Patient-4 belonging to GSVT class. In this example, unlike the previous one, T waves appear negative, while P waves are positive. In the previous example, the signal form focused on both P and T waves, whereas here, it seems to focus exclusively on P waves. This indicates that the general characteristic of the waves being focused on is their positive direction, suggesting that the focus is on waves resembling P waves. This approach is logical in terms of rhythm analysis. Additionally, in this example, it can be observed that the image form focuses along a horizontal trace, which is also a reasonable focal approach.

**Fig. 11:**
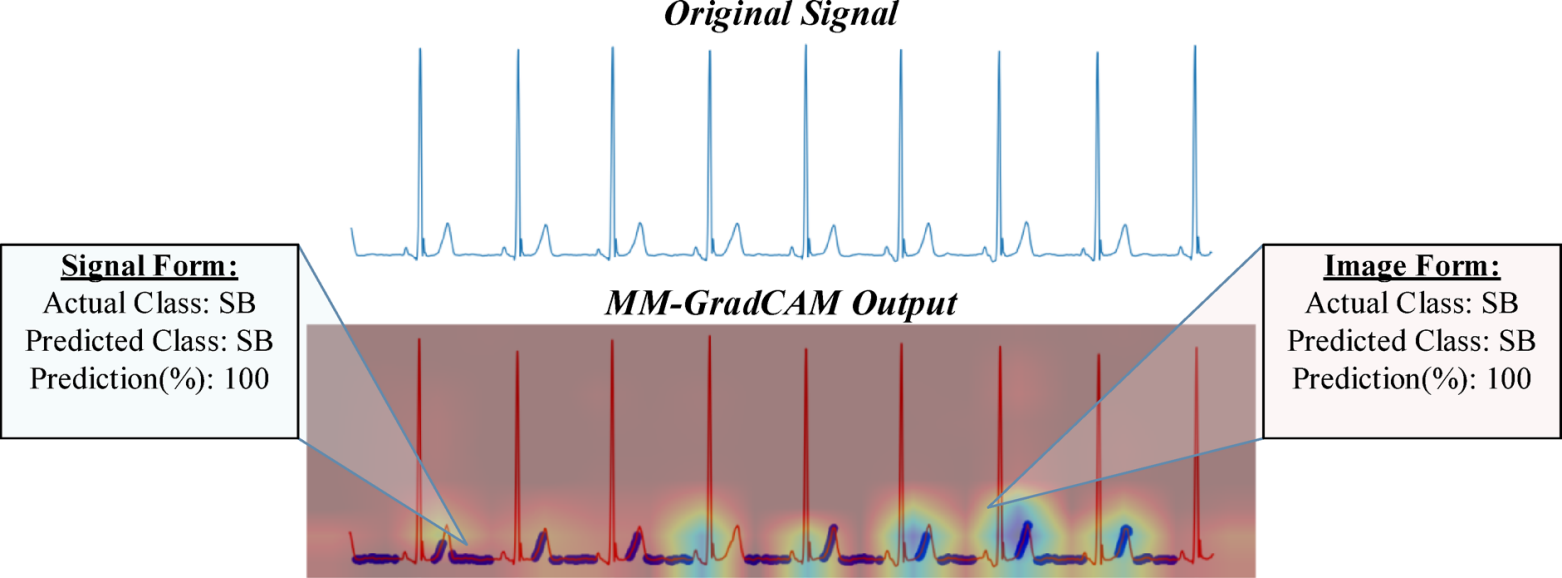
MM-GradCAM output of example Patient-5 belonging to SB class. The signal form is focused consistently on the rising arms of T waves and the T-P intervals, allowing recognition of slow regular rhythm with high accuracy. The image form focuses on T-wave morphology, which also contributes to high accuracy.

**Fig. 12:**
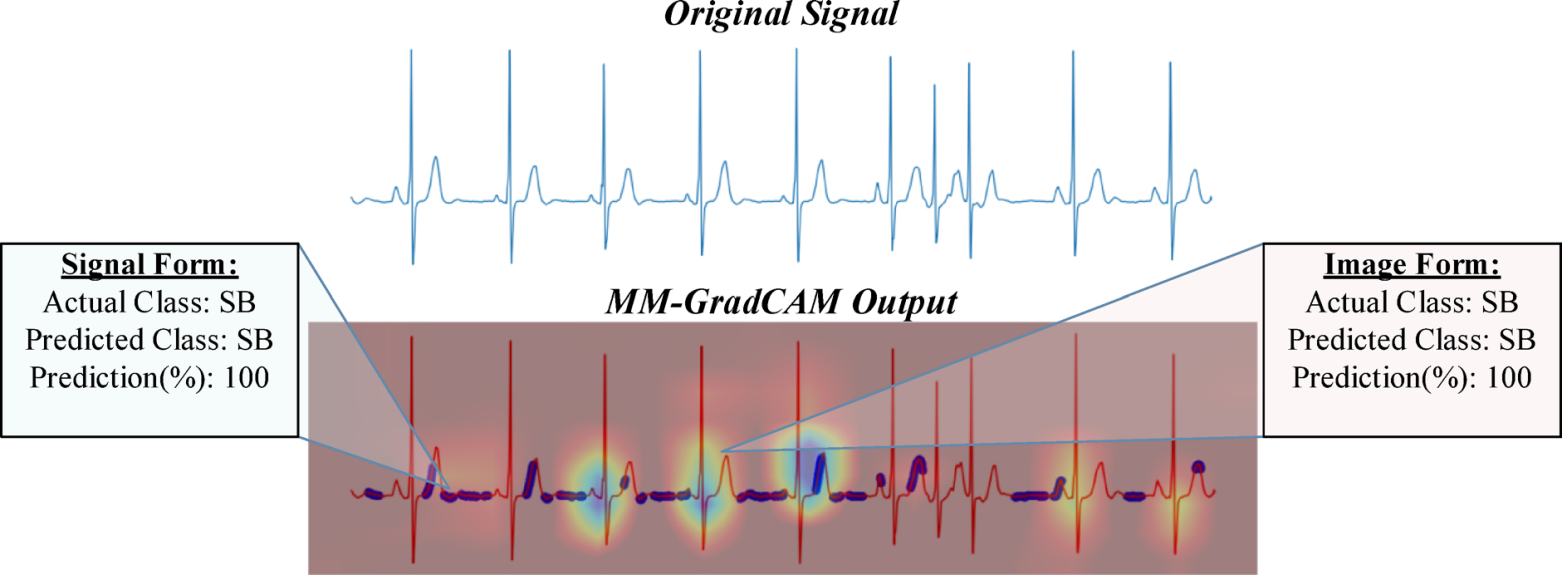
MM-GradCAM output of example Patient-6 belonging to SB class. The signal form focuses on the rising arm of some T waves and most T-P intervals. The image form focuses on QRS complexes. Both forms attain high accuracy despite the presence of atrial extrasystoles, which receive little focus. Here, atrial extrasystoles neither detract from the regions of primary focus nor affect the accuracy of both forms.

**Fig. 13:**
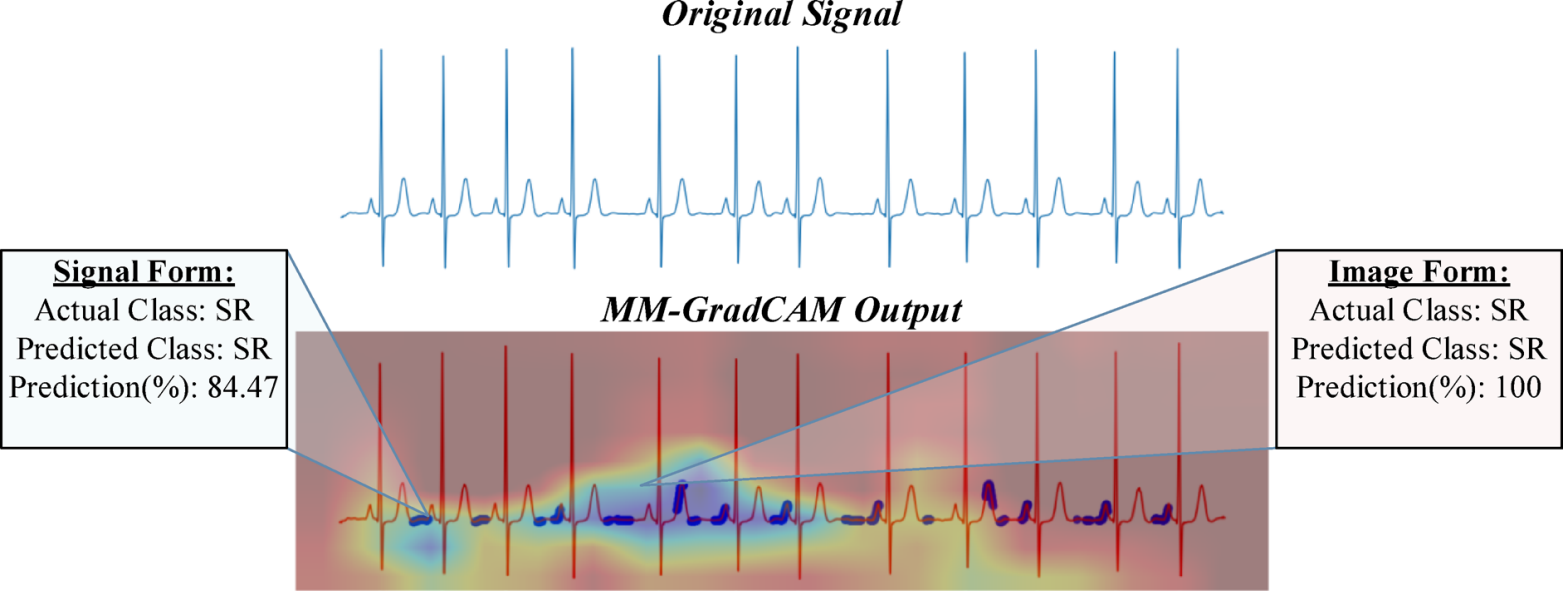
MM-GradCAM output of example Patient-7 belonging to SR class. The ECG shows sinus arrhythmia (or sinus irregularity, which is included in the merged SR class). The signal form focuses mainly on the T-P intervals, which demonstrate irregular periodicity that probably causes the model to suffer in accuracy. The image form focuses on a relatively long segment of consecutive beats that encompasses P and QRS waves, allowing the model to learn the constant relationship between the 2 waves to make a more accurate prediction despite the mild beat-to-beat variation in heart rate.

**Fig. 14:**
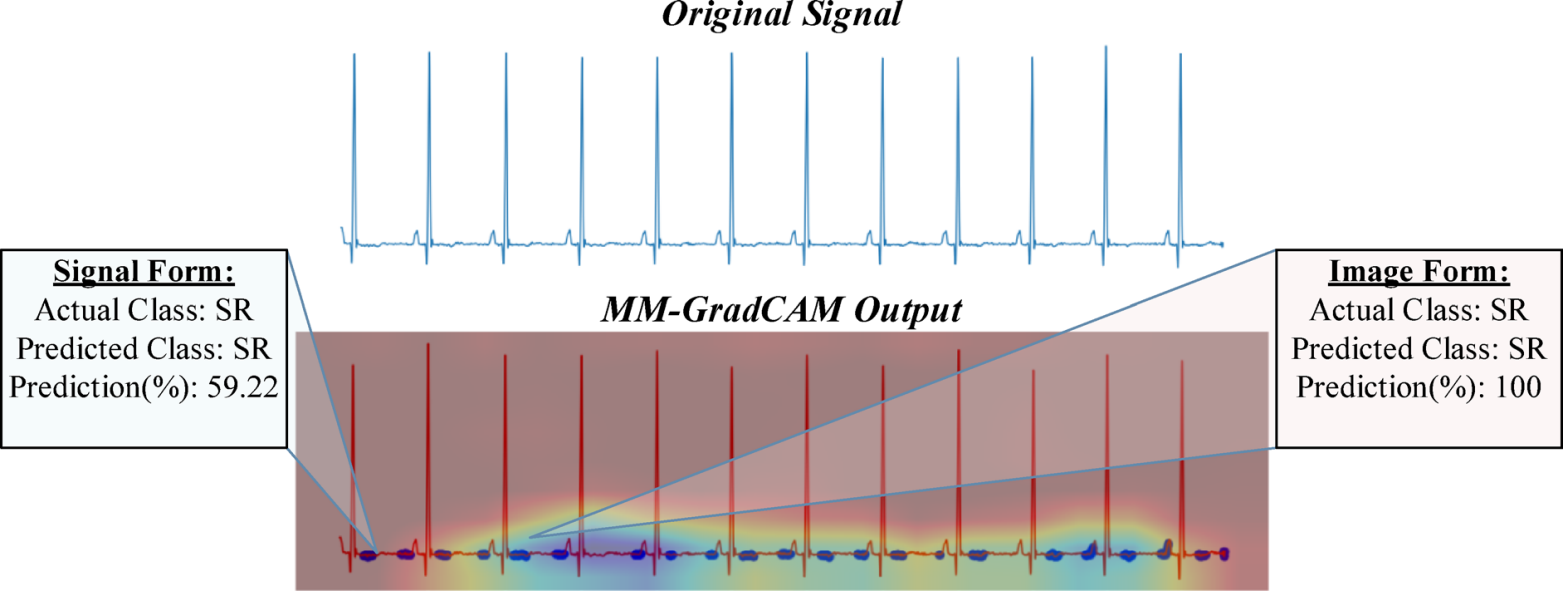
MM-GradCAM output of example Patient-8 belonging to SR class. The ECG shows sinus rhythm with flat and almost indistinguishable T waves. The signal form focuses on discrete T wave and pre-P wave positions, which appear similarly flat and fortuitously equidistant, rendering heart rate and rhythm class predictions inaccurate. The image form focuses on relatively longer segments containing consecutive P and QRS waves, allowing it to assess the sequence of atrial and ventricular depolarizations more reliably, thereby enhancing model accuracy.

In signal form, GradCAM outputs generated based on a specified threshold value are overlayed on the original signal. In image form, the generated heat maps show regions where the model is focused. The two outputs were combined and saved as the final results of the MM-GradCAM method, which were co-registered at the individual patient level with the corresponding softmax outputs. This facilitates expert interpretation, which is important for verifying the explainability obtained in both signal and image forms of the proposed method. Examples of expert evaluation are provided in Figs. 7, 8, 9, 10, 11, 12, 13 and 14. In each of these figures, the original ECG signal, the combined MM-GradCAM output, and the prediction accuracies for the predicted class for both signal and image form models are depicted, allowing the interpreter to visualize the regions that contribute to model predictions for the example and relate this information to model performance.

## Discussion

In this study, raw ECG signals were processed in signal and image forms and trained in parallel on appropriately configured versions of a 17-layer CNN model for arrhythmia detection. The models were trained on a large public 4-class arrhythmia ECG dataset, and the trained models were tested on unseen test data, with explainability maps generated of signal and image forms for individual test patients. To our knowledge, this is the first study in which the explainability of signal and image forms are combined in the same output. In this respect, the study represents an innovation in the field of explainability, contributing to making ECG signals more understandable.

As shown in Table 2, the computational cost of the 2D image-based model is considerably higher than that of the 1D signal model due to the larger input dimensionality and increased memory requirements. To enable practical deployment in clinical environments, several optimization strategies can be applied. These include model pruning to reduce redundant parameters, post-training quantization to decrease model size, lowering image resolution to minimize processing load, and adopting lightweight CNN backbones for faster inference. Additionally, parallel processing and hardware-accelerated execution (e.g., GPU or edge TPU optimization) can further improve runtime performance. Together, these strategies help balance explainability, accuracy, and computational feasibility for real-world clinical implementation.

The use of multimodality provides complementary diagnostic information. While the 1D modality captures fine-grained temporal dynamics of cardiac cycles, the 2D modality highlights spatial and morphological relationships across leads. Each modality has inherent strengths and weaknesses; therefore, combining them allows the model to leverage temporal and spatial cues simultaneously. In several cases confirmed by a cardiologist, the 1D CAM failed to emphasize clinically relevant regions, whereas the 2D CAM successfully identified these patterns. This complementary behavior enhances the robustness and interpretability of the proposed MM-GradCAM, particularly in real-time clinical decision-support applications.

We performed a nonsystematic review of the literature on XAI applications based on ECG diagnosis; the results are summarized in Table 3. It has been observed that more than one lead is generally used in studies carried out to provide clarity in the signal form^16,19,22–26^. In the literature, studies using all 12 leads^22–26^ as well as some researchers have determined the derivations they have chosen to suit their classification purposes^16,19^. For example, Jahmunah et al.^24^ achieved an accuracy of 98.9% between normal and 10 different types of regional involvement in myocardial infarction (MI) using 12-lead ECGs. Similarly, Anand et al.^25^ used 12 leads for MI and arrhythmia detection and achieved AUC values of 93.41% and 99.46%, respectively. Han et al.^26^ performed both 6 different class classifications with 12 leads and achieved 99.85% success in MI localization. Cho et al.^22^, used both 12 and 6 leads for MI detection and obtained higher AUROC values in 6-lead signals. To reduce the data burden of 12 leads, the researchers preferred to focus on specific leads. Wang et al.^16^ performed beat classification with 6 selected leads and achieved 98.64% accuracy. They also extended the study by using a single derivation data from a different data set. Le et al.^19^ classified 13 arrhythmias with only 3 leads and obtained an F1 score of 0.97. Studies focusing on a single lead give researchers the advantage of reducing the data size. Ma et al.^27^ in blood pressure estimation, Kim et al.^17^ used Lead II data in a signal form for arrhythmia prediction. In our study, to focus on only one lead, the most preferred Lead II signals in the literature were used. This choice reduces the data burden and is in accordance with the most common single-lead use in the literature.

**Table 3:** Literature review: XAI studies using ECG signals.

	References	Database	ECG input types	Target	Class	Deep method + explainability	Performance
Signal	Cho et al.^22^	Clinical data	6-lead Signals 12-Lead Signals	MI Detection	Normal and MI	CNN + GradCAM	AUC: 0.880 AUC: 0.854
	Wang et al.^16^	MIT-BIH ADB PTB-XL DB	Lead II Lead I, II, V1, V2, V3, V6	ECG Beat Classification	N, S, V, F, Q	ResNet + GradCAM	Acc: 98.64%
	Hicks et al.^23^	Inter99 DB	12-Lead Signals	Sex Prediction	Male, Female	CNN + GradCAM	Acc: 88.08%
	Ma et al.^27^	MIMIC-III DB	PPG Signals, Lead II Signals	Blood Pressure Prediction	SBP and DBP	AlexNet, GoogLeNet, ResNet18, DenseNet121, DPN68	DenseNet121: MAE: 3.69, 1.95 RMSE: 5.18, 2.71
	Jahmunah et al.^24^	PTB DB	12-Lead Signals	MI Detection	Normal and 10 types of MI	CNN + GradCAM DenseNet + GradCAM	Acc: 98.9%
	Anand et al.^25^	PTB-XL DB Chapman	12-Lead Signals	- MI Detection - Arrhythmia Classification	5 types of MI AFib, GSVT, SB, SR	CNN + SHAP	AUC: 93.41% AUC: 99.46%
	Singh et al.^32^	MIT-BIH ADB	Beat Signals	Arrhythmia Classification	6 types of Arrhythmia	CNN + ECANet + K-GradCAM	Acc: 98.88%
	Kim et al.^17^	Clinical data	Lead II Signals	Arrhythmia Detection	Normal and Arrhythmia	DenseNet + GradCAM	Acc: 98.71%
	Han et al.^26^	PTB DB PTB-XL DB	12-Lead Signals	MI Detection and Localization	Normal and 5 types of MI	ResNet + GradCAM	Acc: 99.97% Acc: 99.85%
	Loh et al.^33^	Clinical data	Segmented Raw ECG Signals	ADHD and CD Detection	ADHD, CD, ADHD + CD	CNN + GradCAM	Acc: 96.04%
	Le et al.^19^	Chapman, CPSC	Lead I, II, V1 ECG Signals	Arrhythmia Classification	13 types of Arrhythmia	1DSEResNet + GradCAM	F1 Score: 0.9718 F1 Score: 0.8004
Image	Makimoto et al.^31^	PTB DB	9-Lead PNG formatted Images	MI Detection	Normal and MI	CNN + GradCAM	Acc: 87.5% ± 11.8%
	Sangha et al.^28^	CODE study PTB-XL DB	12-Lead PNG formatted Images	Multilabel Prediction	Male and 6 types of Arrhythmia	EfficientNet + GradCAM	AUC: 0.99 AUC: 0.97
	Fang et al.^29^	PTB DB PTB-XL DB	12-Lead 3-D ECG Images	MI Detection	Normal and MI	CNN + GradCAM	Acc: 95.65% Acc: 97.23%
	Varandas et al.^20^	MIT-BIH ADB	Lead II, V5 ECG Images	Arrhythmia Detection	Normal and Arrhythmia	ResNet50 + GB GradCAM	Acc: 93.66% Acc: 91.72%
	Kondo et al.^18^	Clinical data	Lead II, V3, V5, aVR ECG Images	CCU Mortality Prediction	CCU Survivors and Non-survivor	CNN + GradCAM	Acc: 77.3%
	Ao et al.^30^	PTB-XL DB, CPSC, Chapman, Tongji	12-Lead ECG Images	Arrhythmia Classification	Diagnoses of 15 types	VGG16 + GradCAM	AUC: 0.982
Image + Signal	Our study	Chapman^21^	Lead II	Arrhythmia Classification	AFib, GSVT, SB, SR	CNN + MM-GradCAM	Signal Form: Acc: 93.07% Image Form: Acc: 97.44%

Acc: Accuracy, Sen: Sensitivity, Spec: Specificity, AUC: area under the receiver operating characteristics curve, N: non-ectopic, S: supraventricular ectopic, V: ventricular ectopic, F: Fusion, Q: Unknown, SBP: Systolic Blood Pressure, DBP: Diastolic Blood Pressure, MAE: Mean Absolute Error, RMSE: Root Mean Square Error, ADHD: Attention Deficit Hyperactivity Disorder, CD: Conduct Disorder, CCU: Cardiac Care Unit.

Studies conducted in image form generally did not focus on a single lead but used multiple leads. For example, Sangha et al.^28^ 12-lead ECG signals into PNG format images, while Fang et al.^29^ 12 leads into 3D ECG images. Ao et al.^30^ obtained an AUROC value of 0.982 in 15 different disease diagnoses with 12-lead images. Studies focussing on specific leads rather than using all leads^18,20,31^ also achieved high success. In our study, since only the Lead II lead was preferred in the signal form, only Lead II was used in the image form to harmonize on the same output. This approach optimized the data load by using a single lead and contributed to the image form, providing consistent interpretability with the signal form.

When the literature is examined among the deep learning models, the CNN structure^18,22–25,29,31–33^ is the most preferred model in both signal and image form. The CNN structure is preferred because the explainability methods can be applied to the last convolution layer, which makes the outputs of the model more understandable. Models such as ResNet and DenseNet, which are extended and multilayer open convolutional versions of CNN^15,17,19,20,24,26–28,30^ It has also been used quite widely. In our study, a CNN model with 17 layers was preferred, considering its suitability for explainability requirements and its widespread use in the literature. This structure provides a strong basis for explainability in signal and image forms.

The most widely used method in XAI approaches is Grad-CAM, and various versions of Grad-CAM have been developed in the literature by adding new perspectives. Singh et al.^32^ proposed a new K-GradCAM interpretability method that updates the weights based on the change in decision probability by combining the advantages of gradient-based backpropagation and perturbation approaches. Similarly, Varandas et al.^20^ developed an innovative approach for Grad-CAM using three different back-propagation-based explainability methods. In this study, a new approach called MM-GradCAM is proposed to contribute to the literature. This method provides a unique interpretation by combining the explainability of signal and image inputs on the same output and makes an innovative contribution to the existing Grad-CAM approaches.

Although previous studies using the same dataset have reported comparable or even higher classification accuracies, the primary objective of the present work is not to solely outperform existing models in terms of numerical performance metrics. Instead, this study focuses on advancing interpretability and clinical relevance through a multimodal learning paradigm. Unlike prior approaches that rely exclusively on 1D ECG signals, the proposed framework jointly exploits 1D temporal information and 2D image-based morphological representations, enabling complementary feature learning that cannot be captured by single-modal models. More importantly, this work introduces a multimodal Grad-CAM–based explainability mechanism specifically tailored for ECG analysis, providing synchronized visual explanations in both signal and image domains. Such dual-domain interpretability facilitates more transparent model behavior and enhances clinician trust, which remains largely unaddressed in earlier studies. Furthermore, the lightweight design of the proposed architecture ensures low memory consumption and fast inference, supporting real-time clinical applicability. Therefore, the significance of the proposed model lies in its ability to deliver an interpretable, generalizable, and clinically meaningful multimodal decision-support framework rather than merely improving accuracy on a well-studied dataset.

### Limitations, mitigation strategies, and clinical implications

Despite the strong performance of the proposed method, several practical limitations should be acknowledged. Mild class imbalance may influence the sensitivity of the model for less represented classes. In addition, ECG recordings may contain noise or artifacts, and atypical or non-standard morphologies may challenge the robustness and interpretability of the model. To reduce these effects, incomplete or corrupted signals were removed and a balanced train–test split was applied. Future work will incorporate additional strategies such as data augmentation, automated artifact detection, and adaptive preprocessing tailored for non-standard ECG presentations to further enhance the reliability and stability of the MM-GradCAM approach.

Although the present study did not include evaluation on an independent external dataset, the proposed MM-GradCAM method is inherently compatible with any ECG dataset containing 1D signal recordings. Therefore, it can be applied to a wide range of existing public and clinical ECG databases. In future work, we aim to validate the method on additional datasets such as PTB-XL, Chapman-Shaoxing, and CPSC to demonstrate its robustness and generalizability across different populations and acquisition settings.

This study did not include a formal quantitative user study involving multiple clinicians, as the primary aim was to introduce the MM-GradCAM method and analyze its explainability outputs. Future work will extend this evaluation by involving a larger group of independent clinicians and collecting quantitative measures such as confidence scores, inter-rater agreement, and task performance metrics to systematically assess the clinical impact of the proposed method.

Beyond its technical performance, the proposed MM-GradCAM method has several potential clinical implications. By simultaneously visualizing salient regions in both the signal and image domains, the approach offers clinicians an intuitive transparent representation of the model’s decision process. This can support rhythm interpretation, increase confidence in automated arrhythmia detection, and facilitate communication in multidisciplinary settings. Moreover, the method may serve as an educational aid for identifying waveform characteristics associated with different arrhythmias. However, real-world deployment will require careful integration into clinical workflows, consideration of variability in ECG acquisition conditions, and validation across broader and more diverse patient groups. Addressing these factors in future studies will be essential to ensure safe and effective clinical translation of the proposed method.

## Conclusion

XAI applications are very important in increasing reliability, especially in the field of health, and encouraging the orientation towards deep learning models. One of the most prominent among these approaches is the Grad-CAM method. Grad-CAM converts the gradient values of the last convolution layer of the model into heat maps and visualises the regions where the model is focused. However, it has been observed in the literature that the Grad-CAM method is generally used with input data in a single format (signal or image).

In this study, MM-GradCAM, a novel approach that combines the explainability of signal and image data, is proposed. In the proposed method, ECG data obtained from a single lead are processed in both signal and image formats. CNN models suitable for these data were designed and Grad-CAM outputs were generated for both formats. The MM-GradCAM method is the first study to combine two different data formats.

Recent studies have been analysed in detail and presented in this paper. The results show that the proposed method performs classification with high accuracy rates. Furthermore, the explainability of both data formats was evaluated by an expert cardiologist and the results were compared. The integration of signal and image formats provides a more comprehensive understanding of the decision-making process of the model and increases reliability in clinical applications. This study paves the way for future research into multimodal explainability techniques, particularly in medical data analysis. It highlights the importance of combining multiple data perspectives to improve model interpretability and reliability in critical areas such as healthcare.

## Data Availability

The dataset utilized in this study originates from the article "A 12-lead electrocardiogram database for arrhythmia research covering more than 10,000 patients" published in Scientific Data (Zheng et al., 2020). This dataset includes electrocardiogram (ECG) recordings of 10,646 patients, annotated with rhythm labels and additional cardiovascular conditions by professional experts. The dataset is publicly available and can be accessed via the figshare repository at: 10.6084/m9.figshare.c.4560497.v2

## Code availability

All source code and implementations for the MM-GradCAM framework (including 1D CNN, 2D CNN and fusion modules) are publicly available on GitHub at https://github.com/FatmaMurat/MM_GradCAM to enable full transparency and reproducibility of our study.
